# Report of the Executive Council of the National Confederation of State Medical Examining and Licensing Boards, at Saint Louis, June 6, 1910

**Published:** 1910-09

**Authors:** N. R. Coleman

**Affiliations:** Chairman; Columbus, Ohio


### Report of the Executive Council of the National Confederation of State Medical Examining and Licensing Boards, at Saint Louis, June 6, 1910.

By N. R. COLEMAN, M.D., Chairman,
Columbus, Ohio.

DURING the past year the duties of the Executive Council have been more onerous than at any time during the history of this organization, but we have labored assiduously and have met and overcome most of the difficulties encountered, and now desire to submit our report for your consideration.

### COMBINING THE NATIONAL AND AMERICAN CONFEDERATIONS.

We first desire to call your attention to a communication which was received on April 9, 1910, from Dr. B. D. Harison, Detroit, Mich., Secretary of the American Confederation of Reciprocating, Examining and Licensing Boards, in Chicago, on or about March 1, 1910, relative to the combining of the American and National Confederations. The American Confederation has appointed a representative of that body to confer with a like representative of the National Confederation.

The Executive Council recommends that a committee be appointed to meet and confer with the committee of the American Confederation, the matter of combining the two organisations to be carefully considered, and a report made to this body during the present session. The National Confederation can, if it desires, instruct its committee relative to the matter. The council believes it to be to the best interests of progress and success in educational and state board work, that there should be but one organisation of this kind in the United States. All the communications which have been received relative to the matter will be delivered to the conference committee when it is organised and ready to discharge its duties.

### PUBLISHING THE PROCEEDINGS.

At the close of the meeting at Atlantic City in 1909, it was deemed important that the proceedings of that meeting should be published, on account of the many valuable papers and discussions embodied therein. With this object in view, an effort was made to secure estimates of the necessary expense of publication and to secure funds for the same. The estimates received were high, and after an earnest effort to obtain funds by subscriptions from a few members of the Confederation, it was found impossible to raise an adequate amount. Therefore, in January, 1910, your chairman requested the secretary to forward to him the estimates received, and within three days he secured a bid that could be met with the funds at our command. This bid was accepted, and on January 28, the copy was placed in the hands of the printer, 1,200 copies being published; and on the second day of March, 1,000 copies were distributed as follows: to all the members of the various boards of the several states, territories, and the District of Columbia; to all the members of the Confederation and collaborators; to all the journals of the state medical societies, and many other journals; and to medical libraries. A letter of invitation to attend the next annual meeting was enclosed with each copy of the proceedings.

PRESS NOTICE.

On April 1, 1910, a press notice of the St. Louis meeting was prepared and sent to the journals, with the request that it be published. Many of them did so, some giving commendatory editorials and all or a part of the program. The Executive Council takes this occasion to cordially thank the various editors for the courtesies extended to the Confederation in so furthering its interests.

ATLANTIC CITY MEETING, 1909.

At the annual meeting of this body, held at Atlantic City, New Jersey, June 7, 1909, there was a symposium on "Practical Examinations Before State Medical Examining Boards." After many valuable papers on the subject had been presented and discussed, it was the consensus of opinion that the present status of practical clinical instruction of medical students did not warrant state boards to attempt practical clinical examinations in many of the important branches of the medical curriculum.

SYMPOSIUM ON CLINICAL TEACHING.

Therefore, it was determined to prepare a symposium on "More Practical and Extended Clinical Instruction of Medical Students." In order to do this, it was thought best to request practical teachers of medicine and surgery to prepare papers upon given themes, which were selected with a view of giving members of state boards the greatest amount of information relative to practical clinical teaching; and in order to bring out various views on practical clinical teaching, it was considered desirable that these subjects should be considered from the viewpoints of the teacher, of the state medical board examiner, and of the profession at large. Accordingly, members from each of these classes have been selected to open the discussions on the papers composing the symposium.

It has required an immense amount of labor to prepare this program, as we were determined to secure none but those who were eminently qualified to prepare papers on the themes given them. It is with much satisfaction that we now submit the result of our effort in the program before you.

### PRINTING AND SENDING OUT PROGRAM AND SECOND LETTER OF INVITATION.

We had printed 1,200 copies of program and 1,000 letters of invitation, of which 800 were distributed on May 18, 1910, as follows: to all members of the various state boards; to members of the Confederation and collaborators; to the deans of the medical colleges; to medical journals; to certain members of the medical profession and others who are interested in educational work along this line.

### PUBLICITY GIVEN TO 1910 MEETING.

We feel warranted in making the assertion that at no time in the entire history of this organisation has such a thorough and wide publicity been given to any annual meeting of this body as to the one in 1910.

### CORRESPONDING SECRETARY.

The managing of the affairs of the National Confederation is far more complex than would appear to one not conversant with conducting its business. The amount of labor necessary, and time consumed, is too much to require of any one person, if the organisation is conducted in the way that it should be to produce the highest and best results. The work is so dissimilar that it cannot be grouped in such a way as to be dispatched rapidly. It should be divided and classified so as to be managed by two persons.

The Executive Council would earnestly recommend that Article V., Section I., of the constitution of the Confederation be amended, and the office of Corresponding Secretary be created, who shall take charge of the papers and transactions of the meetings of the Confederation, prepare the copy for the printer, read the proof, prepare all mailing lists, solicit papers for and prepare the programs, secure persons to discuss papers, prepare press notices, letters of invitation, and all other work of this character relating to the Confederation.

The duty of the Secretary-Treasurer should be to conduct the financial, business, and membership affairs of the organisation. National bodies, like the Confederation, extend over such a vast territory that to send and receive communications from the remote parts requires much time, and unless said communications receive prompt attention, interest in and membership of the organisation will diminish.

### A BRIEF RESUME OF THE WORK DONE BY THE MEDICAL PROFESSION, THE STATE BOARDS AND THE NATIONAL CONFEDERATION.

For the past third of a century the medical profession has been earnestly and assiduously engaged in securing laws for the regulation of the practice of medicine in the United States, and in administering the same. The laws which have been passed are now on trial. Their administration has been largely experimental and pioneer work, as there was no precedent to direct or govern such effort. With these disadvantages it might not be deemed inappropriate here to give a brief résumé of what has been accomplished by the efforts of the National Confederation of State Medical Examining and Licensing Boards alone, in the betterment of medical education in the past twenty years.

In 1899 the report on the minimum standard of preliminary education for the entrance to medical colleges was adopted. It has exerted a very beneficial influence in unifying the minimum educational qualifications for medical students. It was enacted into law in Ohio, and has had an influence in the framing of some of the laws in other states. In 1903 a uniform curriculum was adopted which proved invaluable to medical colleges in making medical education more uniform. At the annual meeting in 1909 the important subject of practical examinations before state medical boards was taken up and considered. There are many other things which have been accomplished by this body that have proven very beneficial to the medical profession, of which the limit of this report precludes mention.

### MEDICAL COLLEGES UNDER STATE CONTROL.

The work of the National Confederation has been almost wholly devoted to work along educational lines, having in view a more thorough education of the physician which would enable him to discharge his duties to those whom he undertakes to serve in the most efficient and successful manner.

In order to accomplish this, it is necessary for the medical colleges to be brought up to, and maintained at, the very highest possible degree of efficiency in equipment and management.

It has been, for many years and is today, believed by teachers and others engaged in educational matters, that this can only be accomplished by placing the medical colleges either directly or indirectly, under state control and by maintaining them, in whole or in part, by endowments or state support, as are the public schools and many of the literary colleges and universities. At the present time there are many medical colleges so controlled and supported, and the results which they attain are of a high order. As long as medical colleges are under the control of individuals and corporations, medical education can never even approximate uniformity, from the fact that when colleges must support themselves, there is more or less rivalry and commercialism entering into their administration, and thereby the time of the teaching faculty is constantly more or less engrossed with the financial and administrative work of the college; whereas, the faculty should be free to devote their entire time to teaching, and this should be wholly separate from the financial concerns of the institution.

All the teachers and professors should receive a just compensation for their time and labor, commensurate with their ability, as do teachers in other educational institutions. In case they fail to perform their duties in an efficient and acceptable manner, they should be removed. There is no valid reason why teachers should give their valuable services without compensation to a medical college, than to any other educational institution. If they do so, it may tend to their own aggrandisement and the detriment of the institution with which they are connected. Moreover, if the institution has to depend upon the patronage which it receives, it is almost impossible to prevent favoritism and the graduating of students who are poorly qualified to receive a degree. There are many other evils which are only too well known by those who have had practical work in colleges, and which could not and would not exist if the colleges were under state control. If under state control, then each student would stand on his own merit, and favoritism and commercialism would not have to be considered. With the brief reasons, which have just been cited, showing why medical colleges should be under state control, the Executive Council, true to its policy of doing pioneer work, would respectfully recommend that at the annual meeting of this body in 1911 a symposium be arranged on the subject of placing all medical colleges, either directly or indirectly, under state control; and that the subject be presented from the viewpoints of the *state, law, medical colleges, the medical profession* and *the state medical examining boards.* It is only by free and varied discussion of such vastly important matters as this that the most information can be secured, the true status determined and the highest and most valuable results attained.

Conducting educational institutions under state control is not, necessarily, an experiment, for it has been for years, and is now, being demonstrated in hundreds of cases that it is feasible to so establish and operate medical colleges and other educational institutions in a most successful manner and with a degree of efficiency higher than can be done in institutions not so controlled.

The most important question to be determined relates to the best methods and means to be employed, whereby the placing of medical colleges under state control can be accomplished. It cannot be done in one year or perhaps in ten years, or by remaining supine and decrying the object in view or the efforts of others to secure the same, but only by an earnest and well directed co-operation; since many laws will have to be amended and, in some cases, new ones enacted. The time required to accomplish this is not a very important factor to be considered when compared with the immense benefits which will necessarily follow when this has been consummated.

In closing this report the Executive Council desires to state its opinion that, by placing the medical colleges under state control, many irregularities and evils which now beset medical education could and would be eradicated, and that uniformity in medical education would be made possible.

The work of upbuilding the medical profession has really but just begun, and should receive the aid and support of every physician who is interested in the welfare of his country and who has at heart the highest development of his chosen profession.

N. R. Coleman, M. D., Chairman.
Edwin B. Harvey, M. D., Boston, Mass.
James A. Duncan, M. D., Toledo, Ohio.
Henry Beates, M. D., Philadelphia, Pa.
D. P. Maddux, M. D., Chester, Pa.
264 E. Town Street.

